# A Decade of Prevalence and Clinicopathological Insights Into Classical Hodgkin Lymphoma: A Study From an Indonesian Tertiary Hospital

**DOI:** 10.7759/cureus.73482

**Published:** 2024-11-11

**Authors:** Agnes S Harahap, Stefanny Charles, Maria F Ham

**Affiliations:** 1 Anatomical Pathology Department, Faculty of Medicine, Dr. Cipto Mangunkusumo/Universitas Indonesia, Jakarta, IDN; 2 Human Cancer Research Center, Indonesian Medical Education and Research Institute, Jakarta, IDN

**Keywords:** classical hodgkin lymphoma, epidemiology, immunohistochemistry, lymphoma, prevalence

## Abstract

Background

Classical Hodgkin lymphoma (cHL) is a lymphoid malignancy originating from germinal center B cells, predominantly affecting young adults. The clinical profile, histologic subtypes, and immunohistochemical (IHC) patterns play crucial roles in diagnosing cHL and predicting prognosis. This study examines the prevalence, clinicopathological features, and IHC patterns of cHL at Dr. Cipto Mangunkusumo Hospital in Jakarta, Indonesia, based on large-scale data collected over a decade.

Methods

This retrospective analysis included 739 confirmed cases of cHL from 2014 to 2023, identified using hematoxylin and eosin staining and an IHC panel. Data on patient demographics, tumor locations, and stages were collected from medical records. The IHC markers utilized were CD20, CD3, CD30, CD15, PAX5, MUM1, Ki67, and CD45. Chi-square and Fisher exact tests were employed to analyze the distribution of subtypes across different age groups and stages.

Result

The most affected age group was 20-29 years, comprising 239 cases (32.34%), with a male predominance of 396 cases (53.59%). The majority of tumors were located in nodal areas, accounting for 532 cases (84.31%), while 99 cases (15.69%) were found in extranodal sites, predominantly in the mediastinum. The nodular sclerosis (NS) subtype was the most frequent, accounting for 461 cases (62.38%), followed by mixed cellularity, lymphocyte-depleted, and lymphocyte-rich classical Hodgkin lymphoma. Most cases were diagnosed at early stages (I-III), with NS significantly associated with early-stage diagnosis (OR 3.06, 95% confidence interval (CI) 1.30-7.22).

Conclusion

The occurrence of HL in terms of prevalence, age, gender, and stage in our study is similar to other Asian countries. Notable correlations were observed between HL subtypes with age and stage.

## Introduction

Hodgkin lymphoma (HL) is a hematological malignancy frequently diagnosed in young adults. This tumor accounts for approximately 80,000 cases and causes 25,000 deaths worldwide annually [1]. There are several risk factors that contribute to HL, such as viral infection, specifically Epstein-Barr virus (EBV), higher socioeconomic status, familial history, and age [2]. HL exhibits a bimodal prevalence pattern, with peaks in the third and fifth decades of life. Additionally, it has been observed that the prevalence of HL increases during the reproductive age [3].

Hodgkin lymphoma arises from germinal center B cells that have lost their ability to transcribe immunoglobulin genes [4]. HL is classified into two main subtypes: classical Hodgkin lymphoma (cHL) and nodular lymphocyte-predominant Hodgkin lymphoma, with the latter being less commonly encountered. The diagnosis of HL can be challenging due to its resemblance to several other types of lymphoma, such as T-cell lymphoma, T-cell/histiocyte-rich large B-cell lymphoma, and occasionally carcinoma. There are significant variations in the therapy and prognosis of the different subtypes of cHL. The four subtypes of cHL are nodular sclerosis, lymphocyte-rich (LRCHL), mixed cellularity, and lymphocyte-depleted (LDCHL). LRCHL generally responds well to current therapeutic approaches, resulting in favorable outcomes. In contrast, LDCHL is often diagnosed at more advanced stages and is associated with a poorer prognosis [5].

To date, there is limited research on the epidemiology and prevalence of cHL in Indonesia. Understanding the prevalence and clinical-pathological profile of HL is crucial for developing targeted healthcare strategies. This knowledge allows for better resource allocation, early diagnosis, and optimized treatment plans tailored to patient demographics. Additionally, it provides valuable insights into disease patterns, enabling public health interventions aimed at reducing morbidity and mortality associated with HL. The objective of this study is to provide a comprehensive analysis of a decade's worth of data on cHL, with a particular emphasis on age and gender distribution, subtypes, and immunohistochemical (IHC) characteristics, at a tertiary referral center in Indonesia.

## Materials and methods

Ethics

This study obtained ethical approval in adherence to the principles outlined in the Declaration of Helsinki. Ethics approval and informed consent waiver were granted by the Institutional Research Ethics Committee of the Faculty of Medicine, Universitas Indonesia-Dr. Cipto Mangunkusumo Hospital (UI-CMH) with letter number KET: 1317/UN2.F1/ETIK/PPM.00.02/2023. Direct informed consent was waived by the committee under permission number ND-2/UN2.F1/ETIK/PPM.00.02/2023.

Sample collection

**Figure 1:**
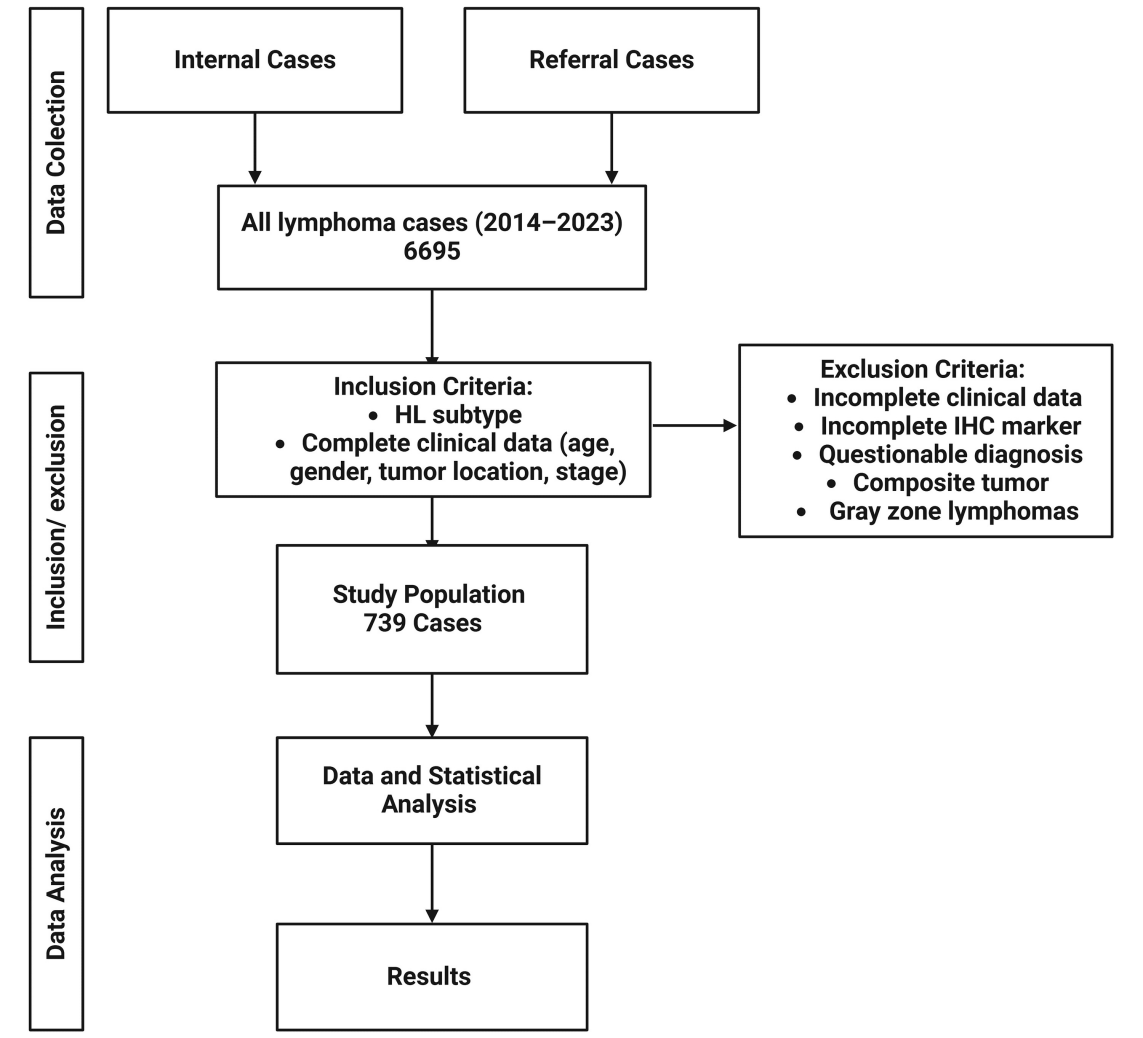
Flowchart of case selection and analysis for Hodgkin lymphoma HL: Hodgkin lymphoma; IHC: immunohistochemical

We conducted a retrospective study on 739 cases of cHL from the Department of Anatomical Pathology, UI-CMH, during the years 2014-2023. The patient population was drawn from in-house patients as well as referrals from affiliated hospitals all over Indonesia (Figure 1). HL diagnoses were confirmed using hematoxylin and eosin (H&E) staining and IHC, performed with the latest World Health Organization (WHO) classification of lymphomas guidelines [6]. Histopathological diagnosis of cHL was based on light microscopy of tissue sections stained with H&E. IHC analysis was performed using a panel of antibodies specific to markers associated with cHL, including CD20, CD3, CD30, CD15, PAX5, MUM1, Ki67, and CD45. IHC staining was conducted using standard protocols recommended by the manufacturers, with appropriate positive and negative controls included in each run. The expression of these markers was independently evaluated by two hematopathologists to ensure diagnostic accuracy. The specific details related to the characteristics of the antibodies are applied in additional information (Table 1). Demographic data, including age, gender, tumor predilection, and stage were extracted from pathology forms required and electronic medical records. Tumor staging was assessed using the Ann Arbor classification system, which categorizes disease stages based on the extent of lymph node involvement and the presence of extranodal disease [7]. Inclusion criteria were confirmed or unequivocal cHL cases with complete clinical data. Cases with incomplete clinical data, incomplete IHC markers, questionable diagnosis, composite tumors, and gray zone lymphomas were excluded from the study.

**Table 1:** Hodgkin lymphoma immunohistochemical panel

Antibody	Clone	Provider	Dilution
CD20	L26	Scytek	1 : 100
CD3	PS1	Biocare	1 : 100
CD30	Ber - H2	Cell marque	1 : 50
CD15	BSB - 119	Biosb	1 : 200
Ki - 67	SP6	Dbs	1 : 50
LCA (CD45)	PD7/26 & 2B11	Scytek	1 : 100
MUM - 1	EAU32	Leica	1 : 200
PAX - 5	BC/24	Biocare	1 : 100

Statistical analysis

Statistical analysis used Microsoft Excel 2023 (Microsoft Corporation, Redmond, Washington, USA) and IBM SPSS Statistics for Windows, Version 26 (Released 2019; IBM Corp., Armonk, New York, United States). Descriptive statistics were used to summarize demographic and clinical data. Categorical variables such as age group, sex, and cHL subtypes were compared using chi-square tests, as appropriate. The relationship between cHL subtypes, patient age, and disease stage was evaluated using odds ratios (OR) and 95% confidence intervals (CI). A p-value of < 0.05 was considered statistically significant.

## Results

**Figure 2:**
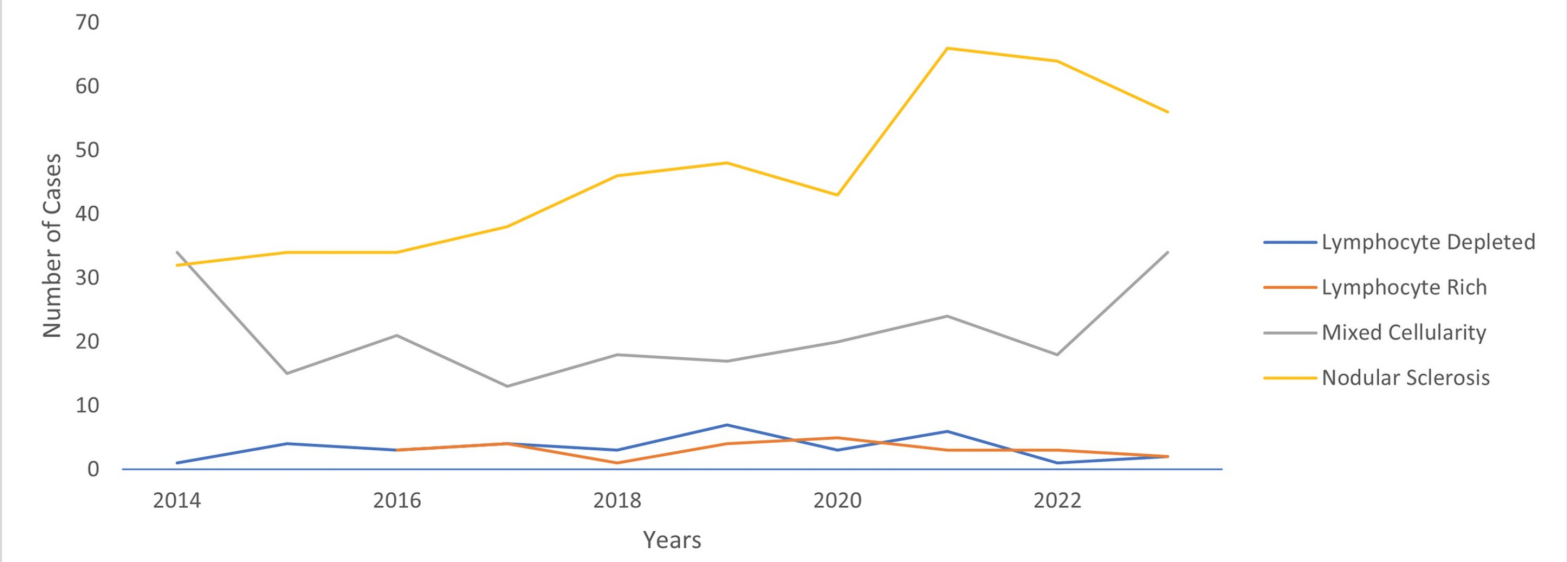
Illustrates ten-year specific subtype patterns of classical Hodgkin lymphoma

Among all lymphoid malignancies identified, 9.1% were classified as HL, with cHL comprising 91.3% of all HL cases. Figure 2 illustrates the yearly distribution of cHL over a ten-year period from 2014 to 2023. The data indicates an ongoing upward trend, although with some fluctuations. The prevalence peaks in 2021.

Table 2 reveals that the most prevalent age group is 20-29 years, comprising 32.34% of the cases. The majority of patients are male (53.59%), with a nodal tumor predilection (84.31%) primarily in the neck/cervical region (75.56%). Among the extranodal cases, the mediastinum is the most common site, representing 70.71%. The nodular sclerosis subtype is the most frequently observed, present in 62.38% of cases, followed by mixed cellularity, LDCHL, and LRCHL. Table 3 presents an analysis of HL subtypes across different age groups.

**Table 2:** Basic demographic of classical Hodgkin lymphoma NOS: not otherwise specified

	Primary cases		Relapse cases	
Characteristics	N	%	N	%
Age				
0 - 9	31	4.19	1	3.57
10 - 19	124	16.78	4	14.29
20 - 29	239	32.34	11	39.29
30 - 39	147	19.89	5	17.86
40 - 49	88	11.91	2	7.14
50 - 59	67	9.07	3	10.71
60 - 69	29	3.92	2	7.14
70 - 79	11	1.49	0	0.00
80 - 89	3	0.41	0	0.00
Sex				
Female	343	46.41	16	57.14
Male	396	53.59	12	42.86
Tumor Predilection				
Nodal	532	84.31	22	91.67
Colli/neck	402	75.56	12	54.55
Supraclavicular	38	7.14	2	9.09
Inguinal	34	6.39	2	9.09
Axilla	25	4.70	4	18.18
Paraaortic	5	0.94	1	4.55
Submandibular	2	0.38	0	0.00
Paravertebral	1	0.19	0	0.00
Multiple nodal locations	25	4.70	1	4.55
Extranodal	99	15.69	2	8.33
Mediastinum	70	70.71	0	0.00
Lungs	7	7.07	0	0.00
Gastrointestinal	4	4.04	0	0.00
Vertebra-pelvis	4	4.04	0	0.00
Lower extremities NOS	3	3.03	1	50.00
Thyroid	2	2.02	0	0.00
Tonsils	2	2.02	1	50.00
Trunk	2	2.02	0	0.00
Upper respiratory tract	2	2.02	0	0.00
Salivary glands	1	1.01	0	0.00
Breasts	1	1.01	0	0.00
Retroperitoneal	1	1.01	0	0.00
Subtypes				
Nodular sclerosis	461	62.38	23	82.14
Mixed cellularity	214	28.96	4	14.29
Lymphocyte rich	30	4.06	0	0.00
Lymphocyte depleted	34	4.60	1	3.57
Stage Ann Arbor (n= 244)				
Stage 1 - 3 (Early)	201	82.38	2	8.33
Stage 4 (Advance)	43	17.62	22	91.67

**Table 3:** Classical Hodgkin lymphoma subtypes analysis within age groups OR: odds ratio; CI: confidence intervals; *chi-square

Subtypes	Age <45	Age ≥45	P-value	OR	95% CI (Lower-Upper)
Nodular sclerosis	395	66	<0.001^*^	2.21	1.35 - 3.21
Mixed cellularity	155	59		0.49	0.33 - 0.71
Lymphocyte rich	24	6		0.94	0.87 - 2.35
Lymphocyte depleted	24	10		0.55	0.26 - 1.17

**Figure 3:**
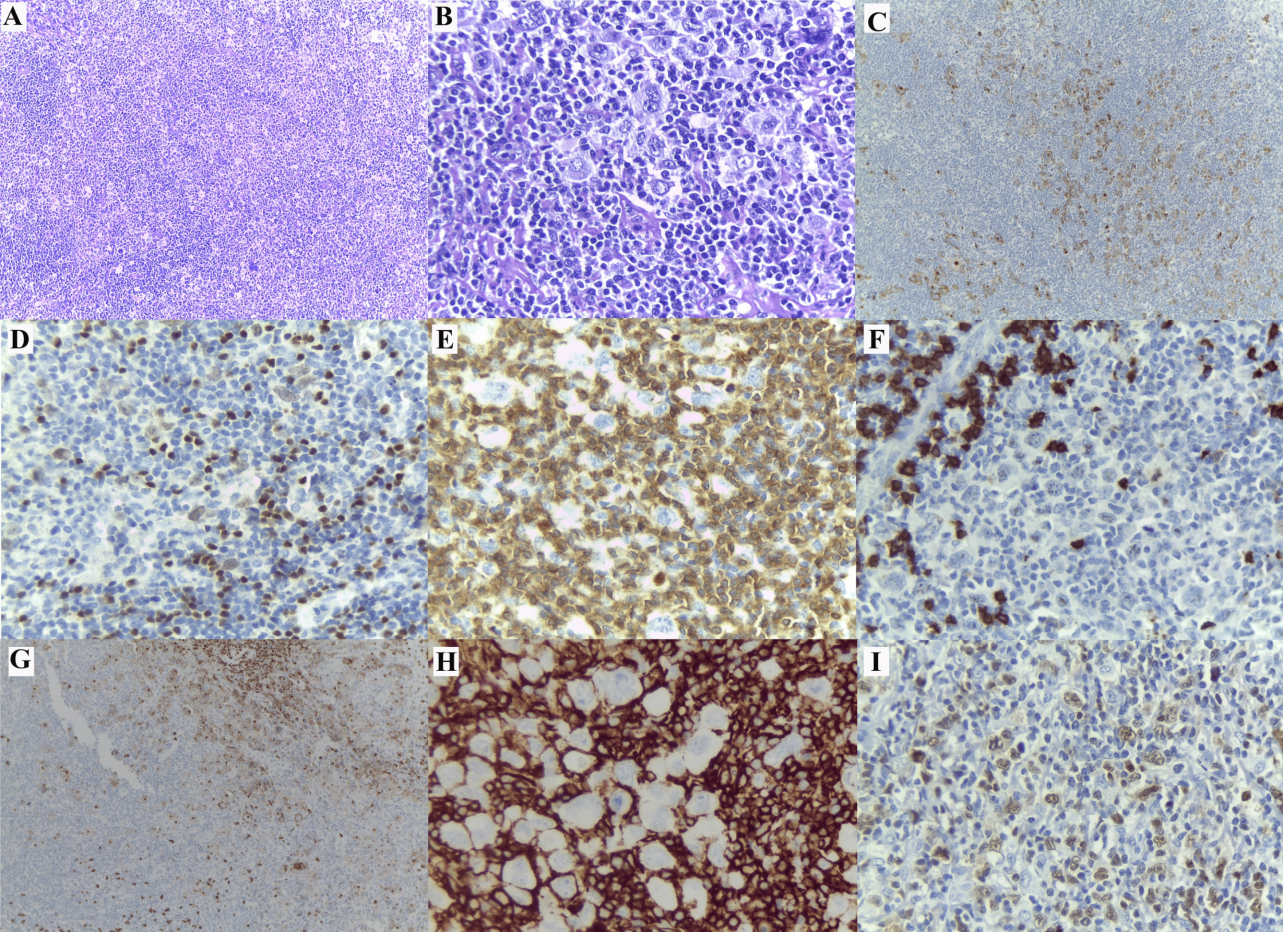
Pathological characteristics of classical Hodgkin lymphoma Classical Hodgkin lymphoma, mixed cellularity subtype, characterized by scattered tumor cells within an inflammatory-rich background (A, H&E, 40x); Reed-Sternberg and mononuclear Hodgkin cells are evident (B, H&E, 400x); Immunohistochemistry shows CD30 positivity with a cell membrane and Golgi pattern (C, IHC, 100x); weak PAX5 positivity (D, IHC, 400x), and CD3-positive small lymphocytes forming rosette-like structures around tumor cells (E, H&E, 400x); CD20 expression is negative (F, H&E, 400x); CD15 expression is positive (G, H&E, 100x); CD45 expression is negative (H, H&E, 400x), MUM1 positive expression (I, H&E, 400x).

The pathological characteristics of cHL are displayed in Figure 3. IHC staining showed positive CD30 expression in all cases, with membranous, cytoplasmic, and Golgi patterns. PAX5 was weakly negative, and MUM1 was strongly positive. The CD20 marker was positive in 196 out of 733 cases (27.03%). Positive CD20 expression was predominantly observed in the nodular sclerosis subtype (15.86%), followed by mixed cellularity (8.69%), LRCHL (1.38%), and LDCHL (1.10%). CD15 was negative in 107 out of 272 cases (39.48%), predominantly in the nodular sclerosis subtype (22.88%), followed by mixed cellularity (11.44%), LDCHL (3.32%), and LRCHL (1.85%).

Approximately 28 cases are known to have experienced a relapse. The predominant age group for relapsed cases was 20-29 years (39.29%), followed by 30-39 years (17.86%), and 10-19 years (14.29%). The gender distribution of relapsed cases was 57.14% female and 42.86% male. Relapse occurred predominantly at nodal sites in 91.67% of cases, with 8.33% occurring at extranodal sites. Regarding subtypes, dominated by nodular sclerosis (82.14%), the CD15 marker was evaluated in 10 out of the 28 relapsed cases, showing negativity in 60% of these cases. Strong positivity for the CD20 marker was observed in 14.29% of cases.

HL cases at CMH from 2018 to 2023 were specifically evaluated for staging. Staging data from referral cases, as well as for cases diagnosed between 2014 and 2017, was unavailable due to incomplete medical records. This study demonstrates a significant association between cHL subtypes and disease stages (p-value 0.032). The nodular sclerosis subtype is more prevalent in the early stages of the disease, with a threefold increased risk. However, the expression of CD15 and CD20 markers does not show a meaningful association with the disease stage (Table 4).

**Table 4:** Analysis of classical Hodgkin lymphoma subtypes, CD15, and CD20 immunohistochemistry markers with tumor stage OR: odds ratio; CI: confidence intervals; *chi-square

	Stage			P-value	OR	95% CI
	Early	Advance				(Lower-Upper)
Subtypes				0.032*		
Nodular sclerosis	126		36		3.06	1.30 - 7.22
Mixed cellularity	55		5		0.31	0.13 - 0.75
Lymphocyte rich	13		0		-	-
Lymphocyte depleted	7		2		1.35	0.27 - 6.74
CD 15				0.508*	1.41	0.51 - 3.93
Negative	34		9			
Positive	48		9			
CD 20				0.070*	0.52	0.25 - 1.06
Negative	161		29			
Positive	40		14			

## Discussion

The prevalence of HL varies significantly between developed and developing countries, with higher frequencies observed in developed regions. In the present study, HL accounted for approximately 9.1% of cases, a prevalence lower than that reported in Western countries and representing 15-20% of all lymphoma cases in these regions. This can be attributed to a combination of factors, including higher rates of immunosuppression, greater exposure to environmental and lifestyle risk factors, and the presence of certain infectious agents [2]. Research indicates that in the United States, Hispanic and non-Hispanic patients exhibit similar overall survival rates despite differences in disease characteristics, such as age at diagnosis [8]. Our study found that the age at diagnosis for HL was younger than typically observed in Western countries, with most cases occurring in individuals under 45 years old. Indonesia is classified as an upper-middle-income country with diverse ethnicities and lifestyles, yet it has limited screening modalities and healthcare resources [9].

Our statistical analysis of cHL cases over the years reveals a consistent upward trend with occasional fluctuations. The annual number of cHL cases demonstrates a progressive increase. Prevalence rates of cHL vary significantly across different countries, with notable surges observed in Southeast Asia and Central and Western Europe, particularly pronounced in Southeast Asian nations [10]. The epidemiological study conducted by Singh et al. predicts a significant 30% rise in cHL cases by the year 2040 [11]. This increase is attributed to cases in the population demographics. In addition, a study conducted by Huang et al. reveals that high-income nations have a greater number of cHL cases, while low-income countries have higher mortality rates. This emphasizes the existence of discrepancies in healthcare access and treatment outcomes [2].

HL exhibits a bimodal age distribution, with peak prevalence observed in individuals aged 15-35 years and a secondary peak in those aged 55 years and older. This study indicates that the highest prevalence of HL is below 45 years of age, with approximately 32.34% of cases occurring in individuals aged 20-29 years. In underdeveloped countries, HL predominantly affects younger individuals and decreases with age, whereas in industrialized nations, the prevalence increases with advancing age [12]. Our study showed that individuals under the age of 45 have a greater probability of developing HL compared to those who are 45 years old or older (p < 0.001). Individuals under 45 years of age have a twofold increased risk of developing the nodular sclerosis subtype compared to those over 45 years of age. Some studies found early age of diagnosis in Asian nations can be linked to smoking, alcohol intake, obesity, and elevated gross domestic product (GDP) per capita [2]. Several studies reported that patients aged 60 years or older constitute approximately 10-20% of the total number of HL cases [13,14]. This finding corroborates the results of this study, which revealed a decreased prevalence of HL in elderly individuals over 60 years old (5.82%).

There are variations in the occurrence of cHL between males and females. Fadhil et al. found that approximately 70% of cHL cases were predominantly male, which supports the findings of this study, where cHL was particularly common in males (53.59%) [15]. Previous research has linked smoking and obesity to increased frequency in women [2]. This study found that the majority of tumors (84.31%) were located in nodal regions, particularly in the neck. These findings are consistent with the studies by Ozuah et al., which identified the neck as the most common site for lymph node tumors [16]. According to the WHO, cervical or supraclavicular lymph nodes are the most common sites for cHL, representing over 70% of cases in the early stages [6]. Meanwhile, our investigation identified supraclavicular as the second most prevalent occurrence at the nodal location. The presence of findings in the supraclavicular region may be associated with lymphatic drainage pathways that facilitate the migration and accumulation of HL cells into the mediastinum [17]. Therefore, the detection of tumors in the supraclavicular area is critical for assessing potential mediastinal involvement [6].

Studies have demonstrated that extranodal manifestations of cHL can affect various anatomical locations, including the digestive system, skin, pleuropulmonary area, bone marrow, thyroid, parotid gland, and breast [8]. In other research, the extranodal area is predominantly identified within the mediastinum region in approximately 68.25% of patients; in this research, we found 70.71%. A study conducted by Sokolov et al. found 60% of extranodal involvement in the mediastinal area in HL patients [18].

Multiple histological subtypes, each presenting specific features, comprise cHL. The most prevalent subtype is nodular sclerosis, characterized by the presence of Hodgkin and Reed-Sternberg cells surrounded by fibrosis and inflammatory cells [19]. Mixed cellularity is characterized by the presence of both a marked inflammatory background and a larger number of tumor cells. This type of cHL is frequently linked to EBV infection [20]. LRCHL can be identified by a dense lymphocytic background and sparse Reed-Sternberg cells [21]. LDCHL is the least common subtype, characterized by a low number of inflammatory cells and diffuse tumor cells. This subtype is associated with advanced-stage disease and has a poorer prognosis [21]. This study indicates that the nodular sclerosis subtype is the most prevalent, accounting for 62% of cases, followed by mixed cellularity, LRCHL, and LDCHL. In contrast, a study from India reported mixed cellularity as the most prevalent subtype, differing from the nodular sclerosis subtype commonly observed in Western populations [22]. According to data from WHO, the nodular sclerosis subtype represented 58.1% of cases, followed by mixed cellularity, LRCHL, and LDCHL [6]. 

IHC examination has been widely used for lymphoma diagnosis. CD30 is a key marker of HL. CD30 expression patterns in Hodgkin lymphoma vary, with distinct localization including paranuclear dot, membrane, and cytoplasmic staining [23]. Standardizing CD30 IHC staining methods is crucial for accurate evaluation and subsequent patient management [24]. CD15 is frequently utilized as an indicator for cHL, and its identification and accuracy might be influenced by different antibodies and staining methods used over time [25]. The presence of this marker has consistently been an important diagnostic indicator for cHL, with positivity rates ranging from 27% to 90%. This is consistent with our findings, where CD15 positivity was observed in 60.52% of cases [26].

Although important, the diagnostic significance of CD20 expression can complicate the diagnosis of cHL. Research indicates that CD20 is present in around 25% of cHL cases, which is consistent with our finding that CD20 positivity was observed in 27.03% of cases [26]. Benharroch et al. reported that approximately 20% of cHL cases express CD20+. This CD20 positivity in cHL is linked to Reed-Sternberg cells, which arise from transformed mature B cells that retain certain B cell markers. Consequently, these cells often exhibit CD20+ or CD30+ expression in cHL [27,28]. The differential diagnosis for this finding includes nodular lymphocytic predominant Hodgkin lymphoma (NLPHL), which is characterized by strong CD20 expression. Notably, NLPHL also demonstrates strong PAX5 expression, lacks MUM1 and CD 30 expression, and is positive for BCL6 with disrupted CD21 architecture. T-cell-rich large B-cell lymphoma can also show a similar morphological pattern and strong expression of CD20. However, CD30 is rarely expressed in this tumor [6]. Furthermore, the presence of EBV infection has been linked to CD20 expression in the NS subtype [29]. CD20 positivity in background cells is related to improved overall survival, especially in patients with large levels of CD20 background cells that compete with tumor-associated macrophages [30]. However, our study showed no association between CD15 and CD20 expression with disease stage. Evaluating the presence of CD15 and CD20, in addition to other markers such as CD30 and PAX5, enhances the precision of diagnosing cHL and assists in differentiating it from other types of lymphomas [31].

This study found that cHL was frequently diagnosed at its initial stages, accounting for approximately 82.38% of all cases. Analysis of the Surveillance, Epidemiology, and End Results (SEER) database in the United States similarly identified that most patients with cHL were diagnosed at stage II. Additionally, Hispanic patients tended to be younger at diagnosis and were more likely to present with B-symptoms compared to non-Hispanic patients [8]. A multicenter study conducted in Italy, Israel, and Spain revealed that 86.3% of patients diagnosed with cHL were classified in stages IIB to IV [32]. In countries such as India, Brazil, and Mexico, 43-53% of cases of cHL are diagnosed at an advanced stage. This prevalence of advanced-stage patients is notably higher in high-income countries [33]. These results highlight the need for region-specific strategies and the impact of socioeconomic factors in the management of cHL.

This current study gives valuable insight into cHL cases at an Indonesian referral hospital, showing a significant pattern in the trend of disease frequency, clinical characteristics, and IHC patterns over the past decade. This research case collects multiple regions; it enhances generalizability and accounts for regional variations. Standardized analysis in line with WHO guidelines offers a comprehensive view of HL that may differ from Western patterns. This study has certain limitations. The incomplete clinical data collection system in our hospital limits our ability to analyze symptoms, relapse status, treatment details, and survival outcomes. Future research is needed to improve these limitations to better understand this disease.

## Conclusions

The prevalence of cHL against age, gender, and disease stage observed in this cohort is consistent with trends documented in other countries. This further highlights the relationship between cHL subtypes with age and disease stage. In addition, IHC markers also support diagnostic accuracy, and we hope that these findings can influence treatment decisions. These findings support more effective resource distribution, earlier diagnosis, and personalized treatment approaches while contributing to public health initiatives aimed at reducing the impact of cHL.
